# Silk Instead of Sponge for Laryngeal Peobangs

**Published:** 1854-01

**Authors:** 


From the Peninsular Journal of Medicine.

*Silk instead of Sponge for Laryngeal Peobangs.* By J. H. B.

Having had occasion to use topical remedies within my own vocal organs, I was surprised at the apparent harshness of the finest sponge I could procure, and was induced to try a ball of silk floss or ravelihgs, well fastened by sewing through-and-through loosely. It holds sufficient of any solution, and does not produce as much involuntary contraction as a sponge; hence it can be passed through the "rima glottidis" in most patients, in the first or second application to the throat, whereas a sponge often requires repeated trials, and is more painful than is necessary.

